# Efficacy and Adverse Event Profile of the iStent and iStent Inject Trabecular Micro-bypass for Open-angle Glaucoma: A Meta-analysis

**DOI:** 10.5005/jp-journals-10008-1248

**Published:** 2018-08-01

**Authors:** Marko Popovic, Xavier Campos-Moller, Hady Saheb, Iqbal Ike K Ahmed

**Affiliations:** 1 Research Assistant, Faculty of Medicine, University of Toronto, Toronto, Canada; 2 Ophthalmologist, Western Health Eye Care Centre, Corner Brook, Newfoundland, Canada; 3 Ophthalmologist, Department of Ophthalmology, McGill University, Montreal, Quebec, Canada; 4 Ophthalmologist, Department of Ophthalmology and Vision Sciences, University of Toronto, Mississauga, Ontario, Canada

**Keywords:** Clinical efficacy, Glaucoma, Meta-analysis, Surgical instruments

## Abstract

**Aim:**

This meta-analysis explores the efficacy and adverse event profile of the iStent, an ab interno implant for the treatment of open-angle glaucoma.

**Methods:**

A systematic literature search of Ovid MEDLINE and EMBASE was used to identify peer-reviewed original studies that provided efficacy data on the first or second generation iStent for at least five eyes. Intraocular pressure (IOP) was the primary efficacy endpoint, while the number of medication classes was the secondary outcome. Weighted mean differences were reported for continuous endpoints, while a relative risk was computed for dichotomous variables.

**Review Results:**

The search revealed 545 results, of which 1767 eyes from 28 studies were included. The cohort age was 71.4 ± 5.4 years, and 44.9% of patients were male. There was a significantly greater IOP reduction after the use of two first-generation stents compared to one, irrespective of phacoemulsification status (p < 0.001). Additionally, there was a significantly greater IOP reduction following iStent alone relative to phaco-iStent for the first-generation iStent (p < 0.001) and the iStent inject (p < 0.001). For the first generation stent, combined phaco-iStent provided a greater level of IOP reduction (p < 0.001) and reduction in the number of medication classes relative to phacoemulsification alone (p < 0.001). In total, 22.5% of eyes that received iStent implantation sustained some type of adverse event. The most common adverse events were intraocular pressure elevation, stent blockage or obstruction, stent malposition and hyphema.

**Conclusion and Clinical Significance:**

Statistically significant differences in efficacy outcomes exist between different numbers of stents and the presence or absence of concurrent phacoemulsification.

**How to cite this article:** Popovic M, Campos-Moller X, Saheb H, Ahmed IIK. Efficacy and Adverse Event Profile of the iStent and iStent Inject Trabecular Micro-bypass for Open-angle Glaucoma: A Meta-analysis. J Curr Glaucoma Pract 2018;12(2):67-84.

## BACKGROUND

Given the irreversible retinal ganglion cell damage resulting from open-angle glaucoma (OAG), current treatment modalities are focused on preserving the structural integrity of the optic nerve and visual function.^1-3^ Prospective evaluations in glaucoma have demonstrated that the reduction of IOP leads to significant sparing of vision: namely, every 1 mm Hg reduction of IOP is correlated with an approximate 10% decrease in the risk of glaucomatous progression.^4^

In OAG, IOP elevation is often a result of reduced aqueous humor flow through the trabecular meshwork^5^ In early stages, ocular hypotensive medications and laser trabeculoplasty have been shown to attenuate glaucoma progression; however there are well known issues with compliance, tolerability, persistence, and difficulty of proper instillation.^3,5^ In the situations in which these treatments are insufficient in reducing IOP to target pressures according to disease severity, ab externo filtering procedures are utilized to provide a more significant IOP reduction. Unfortunately, these techniques are higher risk options that may result in a bleb-related complication, hemorrhage, hyphema, hypotony, infection, inflammation, loss of vision or reoperation.^6,7^

Recently, there has been increasing interest in the ability of microinvasive glaucoma surgery (MIGS) devices to provide a significant level of IOP reduction with less severe postoperative adverse events.^8^ One such device, the iStent ® (Glaukos Corporation, San Clemente, California), is the first ab interno glaucoma implant that has been approved for the management of mild-to-moderate OAG.^9^ The iStent works by allowing aqueous humor to drain directly from the anterior chamber into Schlemm’s canal, thus bypassing a portion of the trabecular meshwork and reducing IOP.^10^ Currently, the iStent has only received food and drug administration approval for use combined with cataract surgery.

Multiple randomized controlled trials and case series have investigated the efficacy and adverse event profile of the iStent device.^2,11-37^ Some have directly compared the combination of iStent implantation and phacoemulsification to phacoemulsification alone.^3,16,17,19-22,30^ Others have been single-armed case series or have compared the iStent to ocular hypotensive medications.^11-15,18,23-29^ More recent research has focused on a second-generation trabecular micro-bypass device termed the iStent inject,^11,14,20,24,29,34,36^ which consists of two heparin coated titanium stents that are both inserted ab interno through the trabecular mesh-work into Schlemm’s canal.^29^ Differences in outcomes between single versus multiple iStents have also been investigated.^11,13,14,17,20,21,23-25,29,31^ In general, most studies have focused on patients with early stages of primary OAG ^11,14-16,21,22,27-29,32^

There has been a rapid expansion of iStent research in recent years.^3,11-37^ Given these new data, it is uncertain whether there are any differences in efficacy between single versus multiple stents or between phaco-iStent compared to either iStent alone or phacoemulsification alone. Additionally, the most frequently reported adverse events in the literature following iStent therapy should be identified. As such, the following meta-analysis aims to investigate the efficacy and adverse event profile of iStent implantation for the management of OAG.

## METHODS

### Literature Search and Data Collection

A systematic literature search was performed on Ovid MEDLINE (2006-Week 1 2018) and Ovid EMBASE (20062018 Week 3). The search strategy that was used can be found in Table 1A and B. Further, Google, Google Scholar and the reference lists of past reviews were manually searched to elicit further relevant literature. Any original prospective or retrospective clinical study that provided relevant efficacy data (i.e., IOP and number of medication classes) on the implantation of the iStent for at least five eyes was included. Only peer-reviewed journal articles were included. Non-english studies, letters to the editor, correspondences, editorials, reviews, opinions, case reports, articles reporting on other surgical procedures and studies that contained repeat data or less than 4 week follow-up were excluded. Studies were screened first by consulting titles and abstracts and afterwards by examining full-text versions. To assist with the screening process, a quality assessment of articles was performed. The Cochrane criteria were used in the assessment of randomized controlled trials, while the National Institute for Health and Care Excellence tool was used to evaluate case series.^38,39^ In both cases, studies were excluded if there was a high risk of bias in at least half of the assessment categories.

**Table 1A:** **Table 1A:** Search strategy for Ovid MEDLINE

*#*		*Searches*		*Results*	
*1*		iStent.m_titl.		29	
2		iStent.mp.		62	
3		Trabecular micro-bypass.mp.		25	
4		Glaukos.mp.		30	
5		Microinvasive glaucoma surgery.mp.		12	
6		Minimally invasive glaucoma surgery.mp.		38	
7		Minimally Invasive Surgical Procedures/		24740	
8		Ophthalmologic Surgical Procedures/		12012	
9		7 and 8		86	
10		Stents/		65102	
11		Glaucoma/		37134	
12		10 and 11		43	
13		1 or 2 or 3 or 4 or 5 or 6 or 9 or 12		222	
14		Limit 13 to yr = “2006-Current”		205	

**Table 1B:** **Table 1B:** Search strategy for Ovid EMBASE

*#*		*Searches*		*Results*	
*1*		iStent.m_titl.		47	
2		iStent.mp.		158	
3		Trabecular micro-bypass.mp.		52	
4		Glaukos.mp.		125	
5		Microinvasive glaucoma surgery.mp.		27	
6		Minimally invasive glaucoma surgery.mp.		73	
7		Minimally invasive surgery/		33752	
8		Eye surgery/		66	
9		1 and 8		66	
10		Stent/		81559	
11		Glaucoma/		51832	
12		10 and 11		87	
13		1 or 2 or 3 or 4 or 5 or 6 or 9 or 12		358	
14		Limit 13 to yr = “2006-Current”		340	

Variables that were included for the baseline demographic evaluation were country of origin, study design, distribution of right and left eyes, age, gender, ethnicity, cup-to-disc ratio, visual field, mean deviation and time of follow-up. The primary efficacy endpoint, IOP, was collected as a continuous variable (i.e., IOP postoperatively and reduction pre- to post-operatively). The postoperative number of hypotensive medication classes and pre- to post-operative reduction in the number of medication classes was the secondary endpoint. For the efficacy analysis, data on the number of iStents and phacoemulsification status (i.e., whether concomitant phacoemulsification was performed) were extracted. For adverse event analysis, the number of events and the four most prevalent events for each study arm were recorded. Postoperative outcomes were collected at last follow-up.

### Statistical Analysis

Weighted mean differences (WMD) and corresponding 95% confidence intervals (95% CI) were reported in the analysis of primary and secondary endpoints. Throughout the analysis, the number of eyes (i.e., sample size) was used as a weighted variable. Alongside a random effects model, the inverse variance method was used in the meta-analysis. The weighted mean was defined as

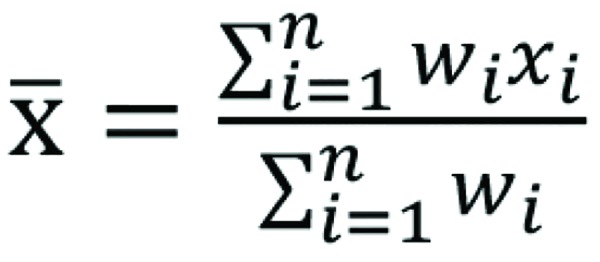



while the weighted standard deviation was computed using the formula

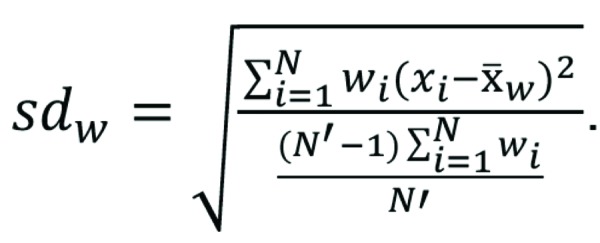



Due to the differential reporting of included studies, each unique endpoint contains data from a different collection of studies. A consequence of this is that the WMDs of IOP and medication class reduction will likely not equal the difference between the preoperative and postoperative values for IOP and medication class count.

In the test for overall effect, a p-value of less than 0.05 was considered statistically significant. The main analysis was performed based on whether patients had 1, 2 or 3 iStents implanted and whether they did or did not receive combined phacoemulsification and iStent. All statistical analyses were performed using Review Manager (RevMan 5.3; The Nordic Cochrane Centre, The Cochrane Collaboration, Copenhagen, Denmark) and Microsoft ® Excel (Microsoft Corporation, Redmond, Washington).

## REVIEW RESULTS

### Study Inclusions and Baseline Demographics

**Fig. 1:**
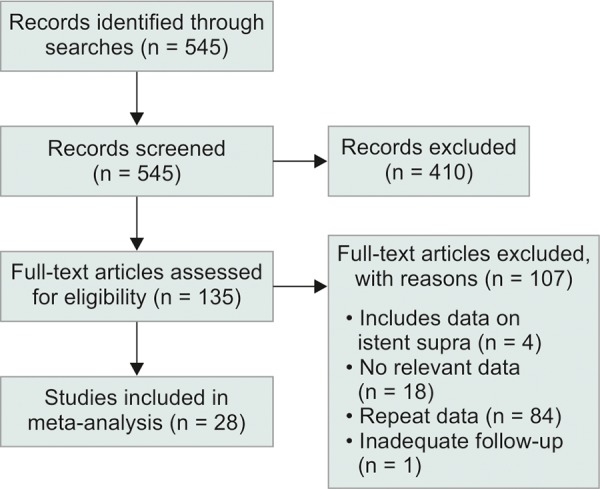
Modified preferred reporting Items for systematic reviews and meta-analysis (PRISMA) flow diagram

The systematic search revealed 545 results. Upon title and abstract screening, the number of potential articles was reduced to 135. Afterwards, full-text screening resulted in 28 studies that met al.l inclusion criteria (Fig. 1).^3,11-37^ Baseline characteristics and the results of quality assessment for included studies are reported on Table 2A. Within the cohort of 1773 eyes for which there was relevant demographic information, the mean age was 71.4 ± 5.4 years (n = 1606; cohort range: 54.4-78.8 years), and 747 out of 1662 eyes were male (44.9%). Most eyes came from Caucasian patients (870 out of 1089 eyes, 79.9%). Generally, studies were moderate to high quality (Tables 2B and C). No study met the a priori condition for exclusion based on the quality assessment.

Of the 1767 eyes included in the efficacy and adverse event analysis, a total of 1217 (68.9%) underwent combined iStent implantation and phacoemulsification, while 497 eyes (28.1%) underwent iStent implantation alone (Table 3). More than half of included eyes had one iStent implanted (999, 56.5%), while 685 eyes had two (38.8%) and 63 eyes received three (3.6%). Overall, the vast majority of eyes (1398, 79.1%) received a first generation iStent, while only 369 eyes (20.9%) received an iStent inject. The distribution of relevant clinical features between groups is presented in Table 4.

In terms of study design, the majority (19/28; 67.9%) of studies were case series, while another 17.9% (5/28) were randomized controlled trials. A total of 60.7% of studies were prospective (17/28), while the rest (11/28, 39.3%) were retrospective. Most studies (22/27; 81.5%) extracted data from a single center while a smaller number were multicentered (5/27; 18.5%).

### Number of iStents-First Generation

Not accounting for phacoemulsification status, meta-analysis was only possible to evaluate the effect of the number of stents on IOP and medication class reduction for first generation iStents (Table 5A-C, Figs 2A and B). When examining IOP reduction, there was a significantly greater decrease after two stents compared to one [WMD = -1.36 mm Hg, 95% CI = (-1.92 mm Hg, -0.80 mm Hg), p < 0.001]. This may have been influenced by the fact that two-stent patients had a significantly greater preopera-tive IOP than one-stent patients [WMD = -1.35 mm Hg, 95% CI = (-1.85 mm Hg, -0.85 mm Hg), p < 0.001]. At the same time, implantation of two stents led to a lesser postoperative IOP when compared to one [WMD = 1.02 mm Hg, 95% CI = (0.80 mm Hg, 1.24 mm Hg), p < 0.001]. There was a greater IOP reduction [WMD= -4.66 mm Hg, 95% CI = (-6.20 mm Hg, -3.12 mm Hg), p < 0.001], higher preoperative IOP [WMD = -2.80 mm Hg, 95% CI = (-3.93 mm Hg, -1.67 mm Hg), p < 0.001] and lower postoperative IOP [WMD = 1.57 mm Hg, 95% CI = (1.12 mm Hg, 2.02 mm Hg), p < 0.001] following three stents relative to one. There was a greater IOP reduction [WMD = -3.30 mm Hg, 95% CI = (-4.93 mm Hg, -1.67 mm Hg), p < 0.001], higher preoperative IOP [WMD = -1.45 mm Hg, 95% CI = (-2.65 mm Hg, -0.25 mm Hg), p = 0.02] and a lower postoperative IOP [WMD = 0.55 mm Hg, 95% CI =(0.06 mm Hg, 1.04 mm Hg), p = 0.03] after three stents relative to two.

**Table 2A:** **Table 2A:** Baseline demographics of included trials

*Study*		*Country*		*Single center or multicenter*		*Study design*		*2016 journal 2-year cites per document*		*Number of eyes*		*Age*		*Number of males*		*Number of Caucasians*		*Mean cup-to-disk ratio*		*Mean visual field (MD, dB)*	
Samuelson et al., 2011		United States		Multicenter		Prospective randomized controlled trial		Ophthalmology; 7.40		117		74 ±8		46		83		n/a		–3.75 ± 3.03	
Fea etal., 2014		Europe		Multicenter		Prospective randomized controlled trial		Clinical ophthalmology; 1.86		94		64.5 ± 10.3		37		94		n/a		n/a	
Buchacra et al., 2011		Spain		Single center		Prospective case series		Clinical ophthalmology; 1.86		10		54.4 ±7.9		9		n/a		n/a		n/a	
Ahmed etal., 2014		Armenia		Single center		Prospective case series		Journal of Cataract and Refractive Surgery; 2.69		39		62.8 ± 12.6		21		39		0.7 ±0.1		–6.47 ± 7.2	
Voskanyan et al., 2014		Europe		Multicenter		Prospective case series		Advances in therapy; 2.98		99		66.4 ± 10.9		43		95		0.7 ±0.2		n/a	
Vandewalle et al., 2009		Belgium		Single center		Prospective case series		Bulletin de la Societe Beige d’Ophtalm ologie; 0.158 (2015)		10		69		n/a		n/a		n/a		–13.7	
Fea, 2010		Italy		Single center		Prospective randomized controlled trial		Journal of Cataract and Refractive Surgery; 2.69		12		64.5 ±3.4		4		n/a		n/a		n/a	
Belovay etal., 2012		Canada		Single center		Prospective case series		Journal of Cataract and Refractive Surgery; 2.69		26		78.8 ± 7		7		18		0.76 ±0.16		–12.6 ±7.1	
2nd study arm		Canada		Single center		Prospective case series		Journal of Cataract and Refractive Surgery; 2.69		23		75 ± 7.3		9		11		0.71 ±0.17		.-10.2 ±8.1	
Patel etal., 2013		United Kingdom		Single center		Prospective case series		Clinical and Experimental Ophthalmology; 2.93		44		76.8		n/a		n/a		n/a		n/a	
Arriola- Villalobos et al., 2012)		Spain		Single center		Prospective case series		British Journal of Ophthalmology; 3.52		19		74.63 ± 8.44		9		19		n/a		n/a	
Arriola-Villalobos et al., 2013		Spain		Single center		Prospective case series		British Journal of Ophthalmology; 3.52		20		75.1 ± 8.6		9		20		n/a		n/a	
Fernandez-Barrientos et al., 2010		Spain		Single center		Prospective randomized controlled trial		Investigative Ophthalmology and Visual Science; 3.15		17		75.2 ±7.2		6		n/a		n/a		n/a	
Spiegel etal., 2009		Europe		Multicenter		Prospective case series		European Journal of Ophthalmology; 1.15		47		76.2 ±6.7		18		46		n/a		n/a	
Wang etal., 2015		Canada		Single center		Retrospective case series		Journal of Ophthalmology; 1.79		96		70.6 ±2.8		53		86		n/a		–7.3 ±2.1	
Klamann etal., 2015		Germany		Single center		Retrospective case series		Graefe’s Archive for Clinical and Experimental Ophthalmology; 2.42		35		61.3 ±3.5		15		n/a		n/a		n/a	
Khan etal., 2015		Canada and United States		Multicenter		Retrospective case series		Journal of Cataract and Refractive Surgery; 2.69		49		77.5 ±11.9		20		34		n/a		–11.5 ±8.0	
Seibold etal., 2016		United States		Single center		Retrospective case series		Journal of Cataract and Refractive Surgery; 2.69		64		73.9 ±8.8		23		34		n/a		n/a	
Gallardo etal., 2016		United States		Single center		Retrospective case series		Clinical Ophthalmology; 1.86		100		74.6 ±8.9		37		14		0.7 ±0.2		n/a	
Ferguson et al., 2016		United States		Single center		Retrospective case series		Clinical Ophthalmology; 1.86		350		74.1 ± 9.0		133		n/a		n/a		n/a	
Lindstrom et al., 2016		Armenia		Single center		Prospective case series		Advances in Therapy; 2.98		57		65.3 ±9.0		30		57		0.7 ±0.1		–4.9 ±5.3	
El Wardani etal., 2015		Switzerland		n/a		Retrospective case series		Klinische Monatsblatter fur Augenheilkunde; 0.52		31		n/a		n/a		n/a		n/a		n/a	
2nd Study Arm		Switzerland		n/a		Retrospective case series		Klinische Monatsblatter fur Augenheilkunde; 0.52		22		n/a		n/a		n/a		n/a		n/a	
Katzetal., 2015		Armenia		Single center		Prospective randomized controlled trial		Clinical Ophthalmology; 1.86		38		68.1 ± 9.1		27		38		0.68 ±0.11		–4.72 ± 4.42	
2nd Study Arm		Armenia		Single center		Prospective randomized controlled trial		Clinical Ophthalmology; 1.86		41		67.8 ±9.3		19		41		0.71 ±0.14		–5.20 ± 5.65	
3rd Study Arm		Armenia		Single center		Prospective randomized controlled trial		Clinical Ophthalmology; 1.86		40		60.9 ±8.1		19		40		0.70 ±0.12		–4.81 ± 4.22	
Shiba etal., 2017		Japan		Single center		Prospective case series		Journal of Ophthalmology; 1.79		10		64.6 ± 10.7		7		0		n/a		–15.4 ±8.1	
Zheng etal., 2017		USA		Single center		Retrospective case series		International Journal of Ophthalmology; 1.30		34		74		9 of 30		21 of 30		n/a		n/a	
Berdahl etal., 2017		Armenia		Single center		Prospective case series		Clinical & Experimental Ophthalmology; 2.93		53		64.7 ±9.6		27		53		0.7 ±0.1		n/a	
Ferguson et al., 2017		USA		Single center		Retrospective case series		Journal of Cataract and Refractive Surgery; 2.69		115		77.42 ±8.51		86		n/a		0.68 ±0.11		n/a	
Gonnermann et al., 2017		Germany		Single center		Retrospective case series		Graefe’s Archivefor Clinical and Experimental Ophthalmology; 2.42		27		73.8 ±7.8		13		27		n/a		n/a	
Kurji etal., 2017		Canada		Single center		Retrospective case series		Canadian Journal of Ophthalmology; 1.57		34		75.02 ± 10.34		11		n/a		n/a		n/a	

*MD = Mean deviation; dB = Decibels; n/a = Not available.

**Table 2B:** **Table 2B:** Quality assessment of included randomized controlled trials (Cochrane criteria)

*Study*		*Year*		*Random sequence generation (Selection bias)*		*Allocation concealment (Selection bias)*		*Blinding of participants and personnel (Performance bias)*		*Blinding of outcome assessment (Detection bias)*		*Incomplete outcome data (Attrition bias)*		*Selective reporting (Reporting bias)*		*Other bias*	
Samuelson et al.		2011		Low		Unclear		High		Low		High		Low		Low	
Fea et al.		2014		Unclear		Unclear		High		High		Low		Low		Low	
Fea		2010		Low		Unclear		Low		Low		Low		Low		Low	
Fernandez-Barrientos et al.		2010		Low		Unclear		Unclear		Low		Low		Low		Low	
Katz et al.		2015		Unclear		Unclear		High		High		Low		Low		Low	

**Table 2C:** **Table 2C:** Quality assessment of included case series (National Institute for Health and Care Excellence Criteria)

*Study*		*Year*		*Multicen-tered*		*Study objective described*		*Inclusion and exclusion criteria reported*		*Outcomes definition reported*		*Prospective*		*Consecutive recruitment*		*Description of study findings*		*Stratification of out-omes*	
Buchacra et al.		2011		No		Yes		Yes		No		Yes		Unclear		Yes		No	
Ahmed et al.		2014		No		Yes		No		Yes		Yes		Unclear		Yes		No	
Voskanyan et al.		2014		Yes		Yes		Yes		Yes		Yes		Unclear		Yes		No	
Vandewalle et al.		2009		No		Yes		Yes		Yes		Yes		Unclear		Yes		No	
Belovay et al.		2012		No		Yes		Yes		No		Yes		Unclear		Yes		No	
Patel et al.		2013		No		Yes		Yes		No		Yes		Unclear		Yes		No	
Arriola- Villalobos et al.		2012		No		Yes		Yes		No		Yes		Unclear		Yes		No	
Arriola- Villalobos et al.		2013		No		Yes		Yes		No		Yes		Yes		Yes		No	
Spigel et al.		2009		Yes		Yes		Yes		No		Yes		Unclear		Yes		No	
Wang et al.		2015		No		Yes		No		Yes		No		Yes		Yes		Yes	
Klamann et al.		2015		No		Yes		Yes		Yes		No		Yes		Yes		No	
Khan et al.		2015		Yes		Yes		Yes		No		No		Unclear		Yes		Yes	
Seibold et al.		2016		No		Yes		Yes		Yes		No		Unclear		Yes		No	
Gallardo et al.		2016		No		Yes		Yes		Yes		No		Yes		Yes		Yes	
Ferguson et al.		2016		No		Yes		Yes		Yes		No		Yes		Yes		Yes	
Lindstrom et al.		2016		No		Yes		Yes		Yes		Yes		Unclear		Yes		No	
El Wardani et al.		2015		No		Yes		Yes		Yes		No		Yes		Yes		Yes	
Shiba et al.		2017		No		Yes		Yes		Yes		Yes		Yes		Yes		No	
Zheng et al.		2017		No		Yes		Yes		No		No		Unclear		Yes		No	
Berdahl et al.		2017		No		Yes		Yes		Yes		Yes		Unclear		Yes		No	
Ferguson et al.		2017		No		No		Yes		Yes		Yes		Yes		Yes		Yes	
Gonnermann et al.		2017		No		Yes		Yes		Yes		No		Unclear		Yes		No	
Kurji et al.		2017		No		Yes		Yes		Yes		No		Yes		Yes		Yes	

**Fig. 2A:**
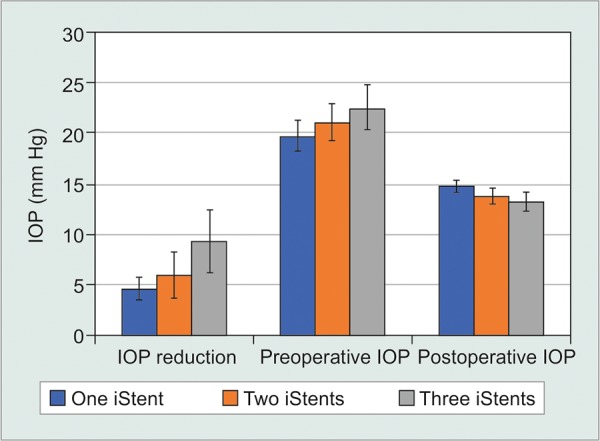
Number of first generation iStents-IOP

**Fig. 2B:**
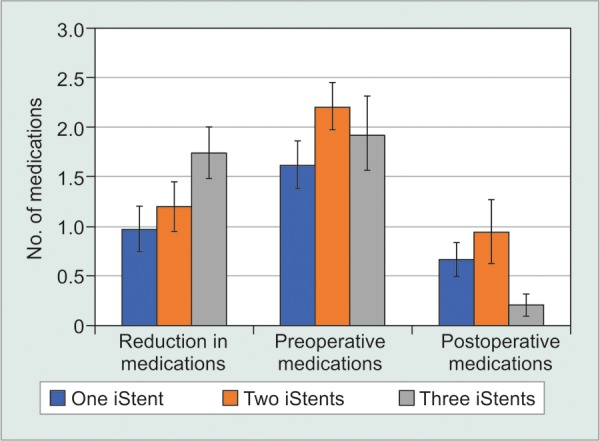
Number of First Generation iStents-number of medication classes

**Table 3:** **Table 3:** Efficacy endpoints and stratification characteristics of included trials

*Study*		*Numbei of Eyes*		*IOP ’ reduction*		*IOP Preoperative*		*IOP Postopera-tively*		*Reduction in medications*		*Number of Medica-tions Preopera-tively*		*Number of Medications Postopera-tively*		*Follow-up (months)*		*Number of iStents*		*Combined Phacoe– mulsification*		*iStent Generation*		*Type of Glaucoma*	
Samuelson et al., 2011		117		8.4± 3.6		25.2 ±3.5		n/a		1.4±0.8		1.5 ±0.7		0.2±0.6		12		1		Yes		First		Any	
Fea et al., 2014		94		12.2± 2.5		25.2 ±1.4		13.0±2.3		n/a		1.0±0		n/a		12		2		No		Second		Primary	
Buchacra et al., 2011		8		6.6±5.4		26.5± 7.9		17.0±2.5		1.1±0.6		2.9±0.7		2		12		1		No		First		Secondary	
Ahmed et al., 2014		39		13.5		25.3 ±1.8		11.8±2.1		1.0±0		2.0±0		1.0±0		18		2		No		First		Any	
Voskanyan et al., 2014		88		10.4±3.2		26.3± 3.5		15.7±3.7		n/a		2.21±0.44		n/a		12		2		No		Second		Pseudoexfol iative	
Vandewalle et al., 2009		9		4.2		20		15.8		1		2.7		1.7		12		1		Mixed		First		Primary	
Fea, 2010		12		3.2±3		17.9± 2.6		14.8±1.2		1.6		2±0.9		0.4±0.7		15		1		Yes		First		Primary	
Belovay et al., 2012		28		3.5		17.3±4		13.8±4		1.8		2.8±0.8		1.0±1.1		12		2		Yes		First		Primary, mixed	
2nd study arm		25		3.9		18.6±4		14.8±3		2.2		2.6±1.2		0.4±0.5		12		3		Yes		First		Primary, mixed	
Patel et al., 2013		44		5		21.5 ±5		16.5±3		1.7		2.3±0.9		0.6±1.0		6		1		Mixed		First		Any	
Arriola Villalobos et al., 2012		19		3.16±3.9		19.42±1.89		16.26±4.23		0.47±0.96		1.32±0.48		0.84±0.89		Mean: 53.68±9.26		1		Yes		First		Any	
Arriola-Villalobos et al., 2013		20		9.42±3		26±3.11		16.75±2.24		1±0.79		1.3±0.66		0.3±0.57		12		1 or 2		Yes		Second		Any open angle	
Fernandez-Barrientos et al., 2010		17		6.6±3.0		24.2±1.8		17.6±2.8		1.1		1.1±0.5		0		12		2		Yes		First		Primary	
Spiegel et al., 2009		42		4.4±4.54		21.7±3.98		17.4±2.99		1.2±0.7		1.6±0.8		0.4±0.62		12		1		Yes		First		Primary	
Wang et al., 2015		96		2.50±5.80		n/a		n/a		1.38±1.43		2.14±0.16		0.76		3		2		Yes		First		Any	
Klamann et al., 2015		32		7.67		22.39±1.81		14.72±0.80		1.3		2.26±0.1		0.96±0.11		6		2		No		Second		Primary, pseudoexfol iative, pigmentary	
Khan et al., 2015		49		n/a		19.6±5.2		14.3±3.1		n/a		2.86±0.91		1.22±1.28		12		2		Yes		First		Primary, pseudoexfol iative, pigmentary	
Seibold et al., 2016		64		1.5		14.7±3.2		13.2±2.8		0.4		1.8±1.1		1.4±1.5		12		1		Yes		First		Any	
Gallardo et al., 2016		134		3.6		16.5±3.7		12.9±2.1		1.4		2.3±1.1		0.9±1.2		12		1		Yes		First		Primary	
Ferguson et al., 2016		350		4.0		19.1±6.3		15.2±3.5		0.6		1.2±1.0		0.6±1.0		24		1		Yes		First		Primary	
Lindstrom et al., 2016		57		10.0		24.4±1.3		14.4±2.1		1.0		1.0±0		0.02		18		2		No		Second		Primary	
El Wardani et al., 2015		31		1.6		16.7		15.1		1.7		2.5		0.8		6		1		Yes		First		N/a	
2nd Study Arm		22		3.2		17		13.8		1.1		2.1		1		6		2		Yes		First		N/a	
Katz et al., 2015		37		10.6		25.0±1.1		14.4 ±1.2		1.6		1.71± 0.61		0.11		12		1		No		First		Primary, pseudoexfol iative, pigmentary	
2nd study arm		41		12.2		25.0±1.7		12.8 ±1.4		1.66		1.76±0.54		0.10		12		2		No		First		Primary, pseudoexfol iative, pigmentary	
3rd study arm		38		12.9		25.1±1.9		12.2 ±1.5		1.43		1.51± 0.69		0.08		12		3		No		First		Primary, pseudoexfol iative, pigmentary	
Shiba et al., 2017		10		5.1		22.0±3.0		16.9 ±3.6		0		3± 0		3±0		6		2		No		First		Primary	
Zheng et al., 2017		17		3		19.7±4.1		16.7 ±2.1		1.4		2.2± 1.2		0.8±1.3		6		1		Yes		First		Any	
Berdahl et al., 2017		53		6.8		19.7±1.5		12.9 ±2.1		1±0		2± 0		1±0		18		2		No		Second		Any	
Ferguson et al., 2017		115		5.49		20.00 ±6.95		14.51 ±2.79		0.7		1.41± 1.04		0.71		24		1		Yes		First		Pseudoexfol iative	
Gonnerman n et al., 2017		25		7.8		21.3±4.1		0. 13.5 ±5		0.72		2.0± 0.9		1.28±1.17		12		2		Yes		Second		Primary, pseudoexfol iative	
Kurji et al., 2017		34		3.87		17.47 ±4.87		13.6 ±3.4		0.32±0.59		2.15± 1.21		1.83±1.2		6		2		yes		First		Primary, pseudoexfol iative	

* IOP = intraocular pressure.

**Table 4:** **Table 4:** Distribution of clinical features for first generation studies by type of analysis

*Type of analysis*		*Baseline feature*		*Comparator 1*		*Comparator 2*		*Proportion of baseline feature in comparator 1 (%)*		*Proportion of baseline feature in comparator 2*	
Number of iStents-reduction in IOP		Phacoemulsification status		One iStent		Two iStents		*iStent alone:* 45/999 (4.5%)		*iStent alone:* 90/287 (31.4%)	
Number of iStents-preoperative IOP		Phacoemulsification status		One iStent		Two iStents		*iStent alone:* 45/999 (4.5%)		*iStent alone:* 90/240 (37.5%)	
Number of iStents-postoperative IOP		Phacoemulsification status		One iStent		Two iStents		*iStent alone:* 45/882 (5.1%)		*iStent alone:* 90/240 (37.5%)	
Number of iStents-reduction in medications		Phacoemulsification status		One iStent		Two iStents		*iStent alone:* 45/999 (4.5%)		*iStent alone:* 90/287 (31.4%)	
Number of iStents-preoperative medications		Phacoemulsification status		One iStent		Two iStents		*iStent alone:* 45/999 (4.5%)		*iStent alone:* 90/336 (26.8%)	
Number of iStents- postoperative medications		Phacoemulsification status		One iStent		Two iStents		*iStent alone:* 45/999 (4.5%)		*iStent alone:* 90/336 (26.8%)	
Number of iStents-reduction in IOP		Phacoemulsification status		One iStent		Three iStents		*iStent alone:* 45/999 (4.5%)		*iStent alone:* 38/63 (60.3%)	
Number of iStents-preoperative IOP		Phacoemulsification status		One iStent		Three iStents		*iStent alone:* 45/999 (4.5%)		*iStent alone:* 38/63 (60.3%)	
Number of iStents-postoperative IOP		Phacoemulsification status		One iStent		Three iStents		*iStent alone:* 45/882 (5.1%)		*iStent alone:* 38/63 (60.3%)	
Number of iStents-reduction in medications		Phacoemulsification status		One iStent		Three iStents		*iStent alone:* 45/999 (4.5%)		*iStent alone:* 38/63 (60.3%)	
Number of iStents-preoperative medications		Phacoemulsification status		One iStent		Three iStents		*iStent alone:* 45/999 (4.5%)		*iStent alone:* 38/63 (60.3%)	
Number of iStents - postoperative medications		Phacoemulsification status		One iStent		Three iStents		*iStent alone:* 45/999 (4.5%)		*iStent alone:* 38/63 (60.3%)	
Number of iStents -reduction in IOP		Phacoemulsification status		Two iStents		Three iStents		*iStent alone:* 90/287 (31.4%)		*iStent alone:* 38/63 (60.3%)	
Number of iStents -preoperative IOP		Phacoemulsification status		Two iStents		Three iStents		*iStent alone:* 90/240 (37.5%)		*iStent alone:* 38/63 (60.3%)	
Number of iStents -postoperative IOP		Phacoemulsification status		Two iStents		Three iStents		*iStent alone:* 90/240 (37.5%)		*iStent alone:* 38/63 (60.3%)	
Number of iStents -reduction in medications		Phacoemulsification status		Two iStents		Three iStents		*iStent alone:* 90/287 (31.4%)		*iStent alone:* 38/63 (60.3%)	
Number of iStents -preoperative medications		Phacoemulsification status		Two iStents		Three iStents		*iStent alone:* 90/336 (26.8%)		*iStent alone:* 38/63 (60.3%)	
Number of iStents - postoperative medications		Phacoemulsification status		Two iStents		Three iStents		*iStent alone:* 90/336 (26.8%)		*iStent alone:* 38/63 (60.3%)	
Phacoemulsification status - IOP reduction		Number of iStents		iStent alone		Phaco-iStent		*One iStent:* 45/173 (26.0%)		*One iStent:* 901/1123 (80.2%)	
Phacoemulsification status - preoperative IOP		Number of iStents		iStent alone		Phaco-iStent		*One iStent:* 45/173 (26.0%)		*One iStent:* 901/1076 (83.7%)	
Phacoemulsification status - postoperative IOP		Number of iStents		iStent alone		Phaco-iStent		*One iStent:* 45/173 (26.0%)		*One iStent:* 784/959 (81.8%)	
Phacoemulsification status - reduction in medications		Number of iStents		iStent alone		Phaco-iStent		*One iStent:* 45/173 (26.0%)		*One iStent:* 901/1123 (80.2%)	
Phacoemulsification status -preoperative medications		Number of iStents		iStent alone		Phaco-iStent		*One iStent:* 45/173 (26.0%)		*One iStent:* 901/1172 (76.9%)	
Phacoemulsification status -postoperative medications		Number of iStents		iStent alone		Phaco-iStent		*One iStent:* 45/173 (26.0%)		*One iStent:* 901/1172 (76.9%)	

IOP = intraocular pressure.

**Table 5A:** **Table 5A:** Efficacy outcomes of one versus two first generation iStent implantation

		*One iStent*						*Two iStents*						*Meta-analysis*							
*Outcome*		*Mean*		*Standard deviation*		*Number of eyes*		*Mean*		*Standard deviation*		*Number of eyes*		*Weighted mean difference*		*95% CI - lower bound*		*95% CI – upper bound*		*p-value*	
IOP reduction		4.67		2.18		999		6.03		4.66		355		–1.36		–1.86		–0.86		p <0.001	
Preoperati ve IOP		19.72		3.06		999		21.07		3.66		240		–1.35		–1.85		–0.85		p <0.0	
Postopera tive IOP		14.80		1.25		882		13.78		1.62		240		1.02		0.80		1.24		p <0.001	
Reduction in medications		0.97		0.46		999		1.20		0.51		287		–0.23		–0.30		–0.16		p <0.001	
Preoperati ve medicatio ns		1.62		0.48		999		2.21		0.48		336		–0.59		–0.65		–0.53		p <0.0 01	
Postopera tive medications		0.67		0.34		999		0.95		0.64		336		–0.28		–0.35		–0.21		p <0.001	

*IOP = Intraocular pressure. CI = Confidence interval

**Table 5B:** **Table 5B:** Efficacy outcomes of one versus three first generation iStent implantation

		*One iStent*						*Three iStents*						*Meta-Analysis*							
*Outcome*		*Mean*		*Standard deviation*		*Number of eyes*		*Mean*		*Standard deviation*		*Number of eyes*		*Weighted mean difference*		*95%CI –Lower bound*		*95%CI –Upper bound*		*p-value*	
IOP reduction Preoperative		4.67		2.18		999		9.33		6.23		63		–4.66		–6.20		–3.12		p <0.001	
IOP Postoperative		19.72		3.06		867		22.52		4.50		63		–2.80		–3.93		–1.67		p <0.001	
IOP Reduction in		14.80		1.25		882		13.23		1.80		63		1.57		1.12		2.02		p <0.001	
medications Preoperative		0.97		0.46		999		1.74		0.53		63		–0.77		–0.90		–0.64		p <0.001	
medications		1.62		0.48		999		1.94		0.75		63		–0.32		–0.51		–0.13		p <0.001	
Postoperative medications		0.67		0.34		999		0.21		0.22		63		0.46		0.40		0.52		p <0.001	

*IOP = Intraocular pressure. CI = Confidence interval. n/a = Not available. Note: red text denotes endpoints that substantially differed from those of the original analysis.

**Table 5C:** **Table 5C:** Efficacy outcomes of two versus three first generation iStent implantation

		*Two iStents*						*Three iStents*						*Meta-Analysis*							
*Outcome*		*Mean*		*Standard deviation*		*Number of eyes*		*Mean*		*Standard deviation*		*Number of eyes*		*Weighted mean difference*		*95%CI –Lower bound*		*95%CI –Upper bound*		*p-value*	
IOP reduction		6.03		4.66		287		9.33		6.23		63		–3.30		–4.93		–1.67		p <0.001	
Preoperative IOP		21.07		3.66		240		22.52		4.50		63		–1.45		–2.65		–0.25		p = 0.02	
Postoperative IOP		13.78		1.62		240		13.23		1.80		63		0.55		0.06		1.04		p = 0.03	
Reduction in medications		1.20		0.51		287		1.74		0.53		63		–0.54		–0.68		–0.40		p <0.001	
Preoperative medications		2.21		0.48		336		1.94		0.75		63		0.27		0.08		0.46		p = 0.006	
Postoperative medications		0.95		0.64		336		0.21		0.22		63		0.74		0.65		0.83		p <0.001	

*IOP = Intraocular pressure. CI = Confidence interval. n/a = Not available. Note: Red text denotes endpoints that substantially differed from those of the original analysis.

For the number of hypotensive medication classes, there was a greater reduction in medication classes following two iStents relative to one [WMD = -0.23, 95% CI = (-0.30, -0.16), p < 0.001]. There was a significantly greater number of medication classes in two stent patients compared to one both preoperatively [WMD = -0.59, 95% CI = (-0.65, -0.53), p < 0.001] and postoperatively [WMD = -0.28, 95% CI = (-0.35, -0.21), p < 0.001]. Comparing between one and three stents, there was a significantly higher number of medication classes [WMD = -0.32, 95%CI = (-0.51, -0.13), p < 0.001] in the three stent cohort preoperatively, as well as a greater reduction in medication class number [WMD = -0.77, 95% CI = (-0.90, -0.64), p < 0.001). Postoperatively, the three stent group had a significantly lower medication class count [WMD = 0.46, 95% CI = (0.40, 0.52), p < 0.001]. There was a greater reduction in medication classes [WMD = -0.54, 95% CI = (-0.68, -0.40), p < 0.001], lower preoperative [WMD = 0.27, 95% CI = (0.08, 0.46), p = 0.006] and lower postoperative medication class count [WMD = 0.74, 95% CI = (0.65, 0.83), p < 0.001] following three stents relative to two.

### Phacoemulsification Status-First Generation

Next, studies were categorized by whether phacoemulsification was performed, irrespective of the number of first-generation iStents (Table 6A, Figs 3A and B). Data revealed that the iStent alone group produced a significantly more pronounced reduction in IOP than the phaco-iStent cohort [WMD = -7.44 mm Hg, 95% CI = (-7.82 mm Hg, -7.06 mm Hg), p < 0.001]. The iStent alone group also had a significantly greater preoperative IOP than the phaco-iStent cohort [WMD = -5.72 mm Hg, 95% CI = (-5.93 mm Hg, -5.51 mm Hg), p < 0.001]. Nonetheless, the iStent alone cohort had a lower postoperative IOP relative to the phaco-iStent cohort [WMD = 1.42 mm Hg, 95% CI = (1.15 mm Hg, 1.69 mm Hg), p < 0.001].

**Table 6A:** **Table 6A:** First Generation iStent - Efficacy Outcomes of Phaco-iStent versus iStent Implantation Alone

		*Phaco-istent*						*Istent implantation alone*						*Meta-analysis*							
*Outcome*		*Mean*		*Standard deviation*		*Number of eyes*		*Mean*		*Standard deviation*		*Number of eyes*		*Weighted mean difference*		*95% CI –Lower bound*		*95% CI –Upper bound*		*P-value*	
IOP reduction Preoperative		4.20		1.82		1123		11.64		2.47		173		–7.44		–7.82		–7.06		p <0.001	
IOP Postoperative		19.27		2.78		1076		24.99		0.88		173		–5.72		–5.93		–5.51		p <0.001	
IOP Reduction in		14.64		1.21		959		13.22		1.72		173		1.42		1.15		1.69		p <0.001	
medications		0.99		0.49		1123		1.33		0.46		173		–0.34		–0.41		–0.27		p <0.001	
Preoperative medications		1.62		0.60		1172		1.87		0.44		173		–0.25		–0.32		–0.18		p <0.001	
Postoperative medications		0.73		0.36		1172		0.55		0.87		173		0.18		0.05		0.31		p = 0.007	

*IOP = Intraocular pressure. CI = Confidence interval. Note: Red text denotes endpoints that substantially differed from those of the original analysis.

Preoperatively, patients receiving combined phaco-iStent were taking significantly fewer medication classes relative to the iStent alone group [WMD = -0.25 mm Hg, 95% CI = (-0.32 mm Hg, -0.18 mm Hg), p < 0.001]. There was a significantly greater reduction in medication class number following iStent alone [WMD=-0.34mmHg, 95% CI = (-0.41 mm Hg, -0.27 mm Hg), p < 0.001] along with a significantly lower postoperative medication class number in the iStent alone arm relative to phaco-iStent [WMD = 0.18 mm Hg, 95% CI = (0.05 mm Hg, 0.31 mm Hg), p = 0.007].

The combination of phacoemulsification and a first generation iStent was also compared to phacoemulsification alone (Table 6B, Figs 4A and B). This comparison only included studies that contained both a phaco-iStent arm and a phacoemulsification alone arm. For this analysis, there was a significantly greater IOP reduction [WMD = 1.68 mm Hg, 95% CI = (1.11 mm Hg, 2.25 mm Hg), p < 0.001] and a higher preoperative IOP [WMD = 2.15 mm Hg, 95% CI = (1.35 mm Hg, 2.95 mm Hg), p < 0.001] following phaco-iStent relative to phacoemulsification alone. However, there was no significant difference between comparators for postoperative IOP (p = 0.07). Phaco-iStent resulted in a significantly more pronounced reduction in medication class number [WMD = 0.80 mm Hg, 95% CI = (0.75 mm Hg, 0.85 mm Hg), p < 0.001] and lower postoperative number of medication classes [WMD = -0.69 mm Hg, 95% CI = (-0.78 mm Hg, -0.60 mm Hg), p < 0.001] relative to phacoemulsification alone. Preoperatively, there was no significant difference between comparators (p = 0.78).

### Phacoemulsification Status-Second Generation

For the second generation iStent *inject,* studies reporting on iStent alone had a significantly greater IOP reduction [WMD = -1.47 mm Hg, 95% CI = (-1.88 mm Hg, -1.06 mm Hg), p < 0.001] and a greater preoperative IOP [WMD = -0.79 mm Hg, 95% CI = (-1.54 mm Hg, -0.04 mm Hg), p = 0.04] compared to studies reporting on phaco-iStent (Table 7, Fig. 5A). Postoperatively, the phaco-iStent cohort had a significantly higher IOP relative to iStent alone [WMD = 0.81 mm Hg, 95% CI = (0.13 mm Hg, 1.49 mm Hg), p < 0.001]. There was a significantly greater reduction in medication classes [WMD=-0.22, 95% CI = (-0.28, -0.16), p < 0.001], higher number of pre-operative medication classes [WMD = 0.20, 95% CI = (0.04, 0.36), p = 0.01] and a lower number of postoperative medication classes [WMD = 0.24, 95% CI = (0.02, 0.46), p = 0.03] following iStent alone relative to phaco-iStent (Fig. 5B).

**Table 6B:** **Table 6B:** First Generation iStent-Efficacy Outcomes of Phaco-iStent versus Phacoemulsification Alone

		*Phaco-istent*						*Phacoemulsification alone*						*Meta-analysis*							
*Outcome*		*Mean*		*Standard deviation*		*Number eyes*		*of Mean*		*Standard deviation*		*Number of eyes*		*Weighted mean difference*		*95%Ci –Lower bound*		*95%Ci –Upper bound*		*P-value*	
IOP reduction		6.30		3.10		199		4.62		3.47		319		1.68		1.11		2.25		p <0.001	
Preoperative IOP		22.44		4.24		199		20.29		4.93		319		2.15		1.35		2.95		p <0.001	
Postoperative IOP		15.23		1.53		82		14.84		1.80		196		0.39		–0.03		0.81		p = 0.07	
Reduction in medications		1.40		0.21		199		0.60		0.36		319		0.80		0.75		0.85		p <0.001	
Preoperative medications		1.72		0.47		199		1.71		0.25		319		0.01		–0.06		0.08		p = 0.78	
Postoperative medications		0.38		0.36		199		1.07		0.63		319		–0.69		–0.78		–0.60		p <0.001	

*IOP = Itraocular pressure. CI = Confidence interval. Note: Red text denotes endpoints that substantially differed from those of the original analysis.

**Fig. 3A:**
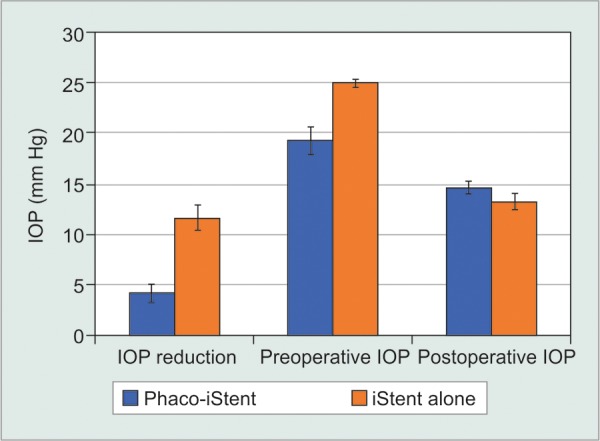
First generation phaco-iStent versus iStent alone-IOP

**Fig. 3B:**
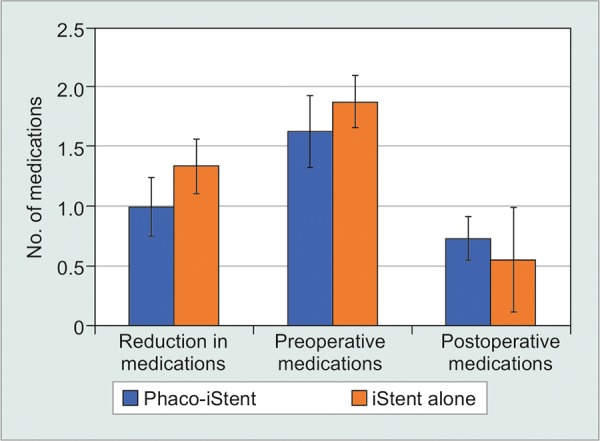
First generation phaco-iStent versus iStent alone-number of medication classes

**Fig. 4A:**
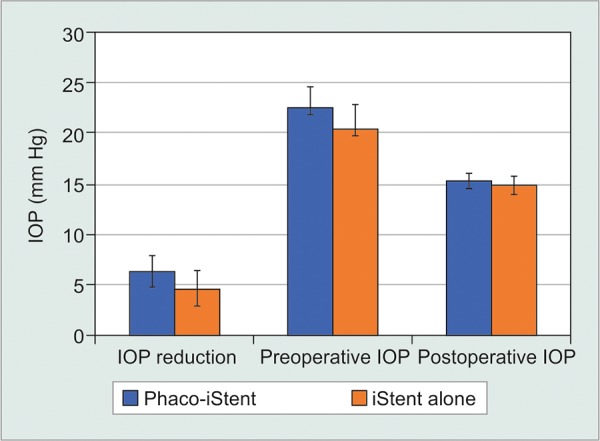
First generation phaco-iStent versus phacoemulsification alone-IOP

**Fig. 4B:**
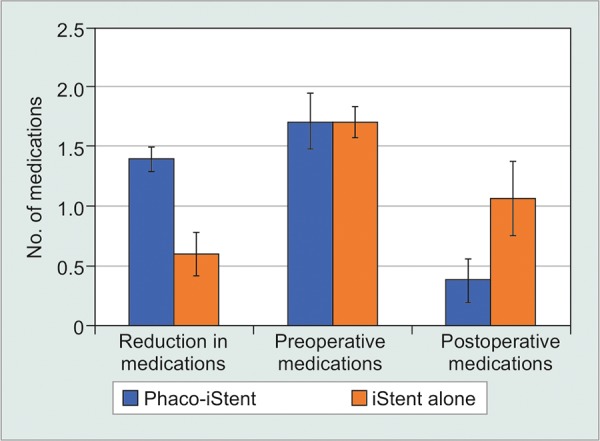
First generation phaco-iStent versus phacoemulsification alone-number of medication classes

### Adverse Event Analysis

Overall, a total of 261 out of 1159 eyes (22.5%) that received iStent implantation sustained some type of adverse event (Table 8). In order from most to least common, the following adverse events were reported: IOP elevation or spike (reported in 12 of 27 papers; 44.4%), stent blockage or obstruction (8/27; 29.6%), stent malposition (7/27; 25.9%), hyphema (6/27; 22.2%), progression of cataract (3/27; 11.1%), blood reflux (3/27; 11.1%), corneal event (3/27; 11.1%), early postoperative event (2/27; 7.4%), stent not visible (2/27; 7.4%), formation of peripheral anterior synechiae (2/27; 7.4%), need for additional surgery (2/27, 7.4%), hypotony (1/27; 3.7%), posterior capsular opacification (1/27; 3.7%), replacement applicator (1/27; 3.7%), patients soreness/discomfort (1/27; 3.7%), transient visual acuity loss (1/27; 3.7%), intraoperative hemorrhage (1/27; 3.7%) and subconjunctival hemorrhage (1/27, 3.7%). Most studies reported either stable or improved visual acuity at last follow-up.

## DISCUSSION

The efficacy and adverse event profile of the iStent device have been explored in a variety of different settings. To evaluate the efficacy and adverse events following iStent implantation based on the consolidation of all peer-reviewed research on the iStent, the present meta-analysis was undertaken.

**Fig. 5A:**
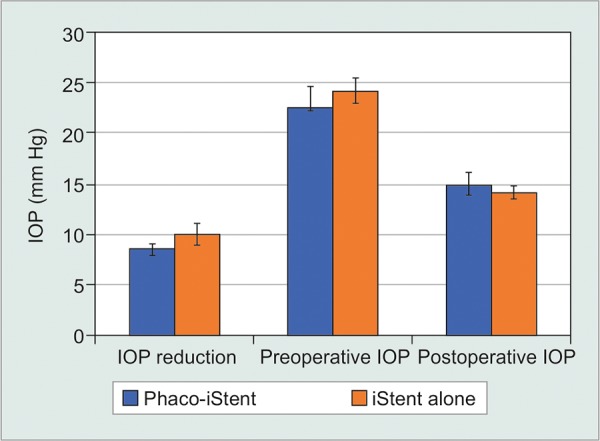
Second generation phaco-iStent versus iStent alone-IOP

**Fig. 5B:**
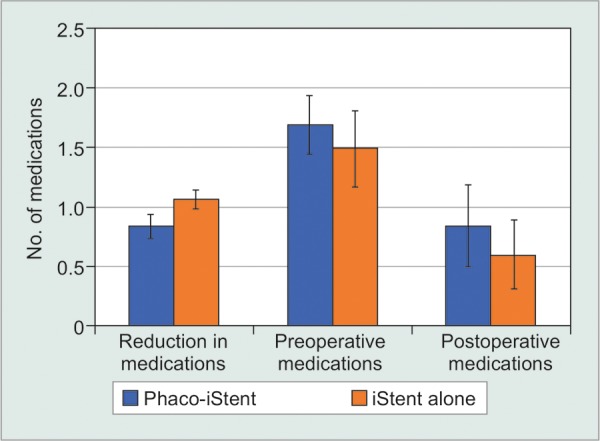
Second generation phaco-iStent versus iStent alone-number of medication classes

In a recent meta-analysis by Malvankar-Mehta et al., the efficacy of the iStent without adjunctive phacoemulsification was analyzed in 248 patients from five studies.^40^ Meta-analysis revealed a significant reduction in IOP after implantation of one [standardized mean difference (SMD) = -1.68, 95% CI = (-2.7, -0.61)], two [SMD = -1.88, 95% CI = (-2.2, -1.56)] and three iStents [SMD = -2, 95%CI = (-2.62, -1.38)]. Glaucoma medication class number was reduced by a mean of 1.2 bottles after one iStent implant, 1.45 bottles after two iStents and one bottle after three iStents.

Another meta-analysis by the same team aimed to investigate the reduction of IOP after phaco-iStent compared to phacoemulsification alone.^41^ A total of 396 patients from 10 studies received phaco-iStent and 1768 patients from 26 studies received phacoemulsification alone. Phaco-iStent produced a significantly greater reduction in IOP relative to cataract extraction alone [SMD = -0.46, 95%CI = (-0.87, -0.06)]. Relative to phacoemulsification alone, phaco-iStent demonstrated a statistically significantly greater reduction in glaucoma medication class number [SMD = -0.65, 95% CI = (-1.18, -0.12)]. Relative to the two studies by Malvankar-Mehta and colleagues, 20 of our 28 included peer reviewed articles have not been reported in previous meta-analyses.^40,41^

The greater IOP reduction with multiple iStents compared to one has been documented in previous laboratory studies and was also confirmed by the findings of the present meta-analysis.^42^ For instance, both postoperative IOP and IOP reduction were significantly improved in the two-stent comparator relative to one. We hypothesize that a selection bias may have influenced these findings, as the higher initial IOP or more severe disease seen in the two-stent comparator may have contributed to the greater IOP reduction following stent implantation. For patients with high preoperative IOP (average of 22.5 mm Hg), three stents provided a more pronounced level of

**Table 7:** **Table 7:** Second generation iStent - efficacy outcomes of phaco-iStent versus iStent implantation alone

		*Phaco-iStent*						*iStent i mplantation Alone*						*Meta-analysis*							
*Outcome*		*Mean*		*Standard deviation*		*Number of eyes*		*Mean*		*Standard deviation*		*Number of eyes*		*Weighted mean difference*		*95% CI –Lower bound*		*95% CI– Upper bound*		*p-value*	
IOP reduction		8.52		1.14		45		9.99		2.14		324		–1.47		–1.88		–1.06		p <0.001	
Preoperative IOP		23.39		2.39		45		24.18		2.53		324		–0.79		–1.54		–0.04		p = 0.04	
Postoperative IOP		14.94		2.28		45		14.13		1.29		324		0.81		0.13		1.49		p = 0.02	
Reduction in medications		0.84		0.20		45		1.06		0.16		142		–0.22		–0.28		–0.16		p <0.001	
Preoperative medications		1.69		0.49		45		1.49		0.64		324		0.20		0.04		0.36		p = 0.01	
Postoperative medications		0.84		0.69		45		0.60		0.58		142		0.24		0.02		0.46		p = 0.03	

*IOP = Intraocular pressure. CI = Confidence interval.

**Table 8:** **Table 8:** Safety endpoints of included trials

*Study*		*Number ofeyes*		*Complications*		*Adverse event 1*		*Adverse event 2*		*Adverse event 3*		*Adverse event 4*		*Visual acuity change*	
Samuelson et al., 2011		111		37		Anticipated early postoperative event		Stent obstruction		Posterior capsular opacification		Stent malposition		97% BCVA improvement	
Fea et al., 2014		94		3		IOP elevation		Soreness/ discomfort		Stent not visible		n/a		Five people experienced decrease	
Buchacra et al., 2011		8		17		Hyphema		IOP elevation		Corneal edema		n/a		No significant change	
Ahmed et al., 2014		39		7		Hypotony		Progression of cataract		Transient visual acuity loss		n/a		CDVA maintained in most eyes	
Voskanyan et al., 2014		88		18		IOP elevation		Stent obstruction		Progression of cataract		Stent not visible		Slight improvement	
Vandewalle et al., 2009		9		10		IOP elevation		Stent malposition		Corneal Erosion		Blood reflux		Stable/improved	
Fea, 2010		12		n/a		n/a		n/a		n/a		n/a		n/a	
Belovay et al., 2012		28		n/a		Stent blockage		Hyphema		Stent malposition		IOP elevation		Stable/improved	
2nd study arm		25		n/a		Stent blockage		Hyphema		Stent Malposition		IOP elevation		Stable/improved	
Patel et al., 2013		44		1		Hyphema		n/a		n/a		n/a		Mean improved	
Arriola-villalobos et al., 2012		19		12		Stent malposition		Stent blockage		Replacement applicator		IOP elevation		Significantly improved	
Arriola-villalobos et al., 2013		20		10		Stent malposition		Stent blockage		Iop elevation		n/a		Significantly improved	
Fernandez-barrientos et al., 2010		17		n/a		Stent malposition		n/a		n/a		n/a		n/a	
Spiegel et al., 2009		42		22		Stent blockage		Stent malposition		Iop elevation		Cataract surgery Complication		Significantly improved	
Wang et al., 2015		96		0		n/a		n/a		n/a		n/a		n/a	
Klamann et al., 2015		32		32		Blood reflux		n/a		n/a		n/a		No decrease	
Khan et al., 2015		49		26		Peripheral anterior synechiae formation		IOP spike		Early postoperative interventions		Hyphema		n/a	
Seibold et al., 2016		64		n/a		n/a		n/a		n/a		n/a		Significant improvement	
Gallardo et al., 2016		134		0		n/a		n/a		n/a		n/a		83% of eyes achieved a BCVA of 20/40 or better after surgery relative to 20% preoperatively	
Ferguson et al., 2016		350		n/a		IOP spike		n/a		n/a		n/a		n/a	
Lindstrom et al.		57		1		Progression of cataract		n/a		n/a		n/a		Stable	
El wardani et al.		31		n/a		n/a		n/a		n/a		n/a		n/a	
2nd study arm		22		n/a		n/a		n/a		n/a		n/a		n/a	
Katz et al.		37		0		n/a		n/a		n/a		n/a		76% of eyes achieved a BCVA of 20/40 or better after surgery relative to 68%	
2nd study arm		41		0		n/a		n/a		n/a		n/a		Preoperatively 66% of eyes achieved a BCVA of 20/40 or better after surgery relative to 61%	
3rd study arm		38		0		n/a		n/a		n/a		n/a		Preoperatively 80% of eyes achieved a BCVA of 20/40 or better after surgery relative to 73% preoperatively	
Shiba et al., 2017		12		Hyphema		Peripheral anterior synechiae		Occlusion by iris		Iop spike		n/a		n/a	
Berdahl et al, 2017		n/a		n/a		n/a		n/a		n/a		n/a		Stable	
Ferguson et al., 2017		8		Iop spike		Need for additional surgery		n/a		n/a		n/a		n/a	
Gonnermann _et_ _al__2017_		29		Reflux _bleeding_		Trabulectomy		n/a		n/a		n/a		n/a	
Kurji et al., 2017		3		Blocked istent		n/a		n/a		n/a		n/a		Approximate 2 line gain on snellen chart	

* BCVA = Best corrected visual acuity; CDVA = Corrected distance visual acuity; IOP = Intraocular pressure.

IOP reduction (9.3 mm Hg) relative to one or two stents. However, interpretations of the three-stent data should be made with caution, as data from only 63 eyes existed for this comparison.

Regardless of the number of implanted iStents, the cohort that underwent first-generation iStent implantation alone saw a more pronounced IOP reduction and lower postoperative IOP than the phaco-iStent group. However, this comparison considers two different patient populations, namely (1) patients receiving iStent alone, who normally do not have cataracts and are receiving the device specifically for IOP reduction, and (2) patients undergoing combined phacoemulsification and iStent, who are receiving the treatment for both their cataracts and an elevated IOP. As such, the finding of a higher preoperative IOP in the iStent alone group may have influenced the difference in IOP reduction between comparators. Even though some included studies contained both patients who received phaco-iStent and iStent alone, subgroup analysis analyzing the differences in outcomes between these two groups was never performed in individual studies.^15,18^ As such, the conclusions derived from comparing phaco-iStent versus iStent alone have not been previously established.

Analysis of phaco-iStent compared to phacoemul-sification alone revealed that there was a greater IOP reduction following phaco-iStent relative to phaco-emulsification alone. This aligns with the findings of Malvankar-Mehta et al., who also showed that there was a significantly greater IOP reduction following phaco-iStent relative to phacoemulsification alone [SMD = -0.46, 95% CI = (0.87, -0.06)].^41^ Despite the similarity, it is important to note that uncontrolled, one-armed studies examining the efficacy of phacoemulsification alone were included in the previous analysis but were excluded in the present article.^41^ Instead, we limited our analysis of phaco-iStent versus phacoemulsification only to the studies that had a phaco-iStent arm and a phacoemulsification only comparator, thus resulting in a more controlled analysis. Beyond analysis of IOP, both meta-analyses concluded that phaco-iStent was statistically superior relative to phacoemulsification alone in the reduction of medication class number pre- to post-operatively.

The adverse event analysis revealed that fewer than 25% of eyes carried some type of adverse event postoperatively, most of which were not serious nor visually threatening. This compares favorably with the postoperative adverse event rates of both trabeculec-tomy and the Baerveldt glaucoma implant.^43^ However, due to differential reporting of adverse events between individual studies, caution should be used when interpreting these findings. In our cohort, IOP elevation, stent blockage or obstruction, stent malposition and hyphema were the most common adverse events following iStent implantation.

Beyond the efficacy and adverse event profile, the cost-effectiveness of the iStent relative to topical glaucoma medications has been studied by Iordanous and colleagues.^44^ Following implantation of two iStents, the authors analyzed cost differences at 6 years postop-eratively. At 6 years, the iStent was $20.77 more expensive relative to monodrug therapy but was cheaper by $1272.55 compared to bidrug treatment and $2124.71 versus tridrug therapy. The authors concluded that the iStent may offer a modest cost saving when compared to glaucoma medications.

Given that past meta-analyses included lower numbers of eyes receiving iStent implantation (first article: 5 studies, n = 248; second article: 10 studies, n = 396), the present work (28 studies, n = 1767) represents the largest quantitative synthesis of efficacy and adverse event data for the iStent device.^40,41^ The large statistical power provided by such a high sample size allowed us to conduct certain analyses that were novel to the published literature; for example, an analysis comparing phaco-iStent to iStent alone. We only included published articles, thus ensuring that the rigors of peer-review were met for each included study.

Limitations of the analysis include the fact that there was no restriction of studies based on design. As such, baseline values for included endpoints were significantly different between comparator arms. As shown in Table 4, the relevant clinical features were often not balanced between groups. As noted by Kaplowitz et al., variation in study design and implementation such as length of follow-up, etiology of disease and baseline clinical indicators may account for the high degree of heterogeneity upon meta-analysis.^45^ Further, since some articles did not include sociodemographic and clinical characteristics of their study cohorts (e.g. surgeon experience), it is uncertain whether there was a balance of these factors between comparator arms. For instance, there

is variable reporting of surgeon experience in the literature: two articles^19,20^ noted that the study surgeon was in an early stage in the learning curve, one noted that the data incorporate the surgeon learning curve,^3^ and another hypothesized how the learning curve influenced the greater number of adverse events in an initial set of patients.^22^ Two studies reported that their surgeons were experienced,^24,30^ while another found no significant difference in outcomes between initial and late procedures.^28^ Another limitation was that the lack of available studies prevented us from performing a robust meta-analysis for some endpoints, such as IOP reduction following three stents, where there was only 63 included patients. Limited reporting of adverse event severity across studies prevented us from analyzing severity in the adverse event analysis. Studies were variable in how they handled medication washout before stent implantation, which made it impossible to analyze the effect of preoperative medications on baseline IOP. Given that data was extracted from study cohorts, conclusions should be limited to the level of the cohort.

## CONCLUSION AND CLINICAL SIGNIFICANCE

The following meta-analysis has shown that there may be differences in treatment response for the iStent due to varying parameters, including the number of iStents and phaco-iStent compared to either iStent alone or phaco-emulsification alone. In our analysis, two stents delivered a greater response in terms of IOP reduction relative to one and iStent alone had a significantly greater IOP reduction compared to phaco-iStent. Combined phaco-iStent was statistically superior relative to phacoemulsification alone in the reduction of IOP and medication classes pre-to post-operatively. Future research should determine whether similar conclusions are reached following meta-analysis in a more controlled environment.

## ETHICAL APPROVAL

This article does not contain any studies with human participants or animals performed by any of the authors. As such, there was no informed consent process needed for this study.

## AUTHORSHIP CONTRIBUTIONS

- *Conception and design of study:* M.P., X.C.M., I.I.K.A.
- *Acquisition of data:* M.P., X.C.M.
- *Analysis and interpretation of data:* M.P., X.C.M., H.S., I.I.K.A.
- *Drafting and revising article:* M.P., X.C.M., H.S., I.I.K.A.
- *Final approval of the version to be submitted:* M.P., X.C.M., H.S., I.I.K.A.
